# Transgenic Monkey Model of the Polyglutamine Diseases Recapitulating Progressive Neurological Symptoms

**DOI:** 10.1523/ENEURO.0250-16.2017

**Published:** 2017-03-28

**Authors:** Ikuo Tomioka, Hidetoshi Ishibashi, Eiko N. Minakawa, Hideyuki H. Motohashi, Osamu Takayama, Yuko Saito, H. Akiko Popiel, Sandra Puentes, Kensuke Owari, Terumi Nakatani, Naotake Nogami, Kazuhiro Yamamoto, Satoru Noguchi, Takahiro Yonekawa, Yoko Tanaka, Naoko Fujita, Hikaru Suzuki, Hisae Kikuchi, Shu Aizawa, Seiichi Nagano, Daisuke Yamada, Ichizo Nishino, Noritaka Ichinohe, Keiji Wada, Shinichi Kohsaka, Yoshitaka Nagai, Kazuhiko Seki

**Affiliations:** 1 Department of Neurophysiology, National Institute of Neuroscience National Center of Neurology and Psychiatry, Tokyo 187-8502, Japan; 2 Department of Degenerative Neurological Diseases, National Institute of Neuroscience National Center of Neurology and Psychiatry, Tokyo 187-8502, Japan; 3 Department of Pathology and Laboratory Medicine, National Institute of Neuroscience National Center of Neurology and Psychiatry, Tokyo 187-8502, Japan; 4 Division of Laboratory Animals Resources, National Institute of Neuroscience National Center of Neurology and Psychiatry, Tokyo 187-8502, Japan; 5 Department of Neuromuscular Research, National Institute of Neuroscience National Center of Neurology and Psychiatry, Tokyo 187-8502, Japan; 6 Department of Peripheral Nervous System Research, National Institute of Neuroscience National Center of Neurology and Psychiatry, Tokyo 187-8502, Japan; 7 Department of Ultrastructural Research, National Institute of Neuroscience National Center of Neurology and Psychiatry, Tokyo 187-8502, Japan; 8 Department of Neurochemistry, National Institute of Neuroscience National Center of Neurology and Psychiatry, Tokyo 187-8502, Japan

**Keywords:** Neurodegenerative disease, Polyglutamine disease, Transgenic monkey, Common marmoset

## Abstract

Age-associated neurodegenerative diseases, such as Alzheimer’s disease, Parkinson’s disease, and the polyglutamine (polyQ) diseases, are becoming prevalent as a consequence of elongation of the human lifespan. Although various rodent models have been developed to study and overcome these diseases, they have limitations in their translational research utility owing to differences from humans in brain structure and function and in drug metabolism. Here, we generated a transgenic marmoset model of the polyQ diseases, showing progressive neurological symptoms including motor impairment. Seven transgenic marmosets were produced by lentiviral introduction of the human ataxin 3 gene with 120 CAG repeats encoding an expanded polyQ stretch. Although all offspring showed no neurological symptoms at birth, three marmosets with higher transgene expression developed neurological symptoms of varying degrees at 3–4 months after birth, followed by gradual decreases in body weight gain, spontaneous activity, and grip strength, indicating time-dependent disease progression. Pathological examinations revealed neurodegeneration and intranuclear polyQ protein inclusions accompanied by gliosis, which recapitulate the neuropathological features of polyQ disease patients. Consistent with neuronal loss in the cerebellum, brain MRI analyses in one living symptomatic marmoset detected enlargement of the fourth ventricle, which suggests cerebellar atrophy. Notably, successful germline transgene transmission was confirmed in the second-generation offspring derived from the symptomatic transgenic marmoset gamete. Because the accumulation of abnormal proteins is a shared pathomechanism among various neurodegenerative diseases, we suggest that this new marmoset model will contribute toward elucidating the pathomechanisms of and developing clinically applicable therapies for neurodegenerative diseases.

## Significance Statement

Various neurodegenerative diseases, including Alzheimer’s disease, Parkinson’s disease, and the polyglutamine diseases, share a common pathomechanism via the abnormal accumulation of misfolded proteins in the central nervous system. Here, we have successfully established a transgenic marmoset model of the polyglutamine diseases, which recapitulates the human disease process including the accumulation of misfolded proteins and neuronal cell loss, resulting in neurologic symptoms. Because the neurologic symptoms initiated at 3–4 months after birth followed by gradual progression, our model opens a time window in future translational research to develop clinically relevant biomarkers and evaluate the preclinical efficacy of therapeutic candidates, making the most of the similarity of both brain structure and function, as well as drug metabolism, between humans and primates.

## Introduction

Modeling human diseases in experimental animals via genetic engineering is an indispensable step in studying the pathomechanisms of and developing therapies for intractable neurodegenerative diseases such as Alzheimer’s disease (AD), Parkinson’s disease (PD), Huntington’s disease (HD), and the spinocerebellar ataxias (SCAs). Indeed, various transgenic and knockout/knockin mouse models of these neurodegenerative diseases have been developed and have substantially contributed to the understanding of basic disease mechanisms (Wong et al. 2002; Rockenstein et al. 2007). However, rodent models have limitations in translational research in terms of evaluating the therapeutic efficacy and metabolic profiles of drug candidates, as well as in developing disease biomarkers, mainly because of the differences in metabolism between humans and rodents (Chan, 2013; Kishi et al. 2014). Furthermore, differences between humans and rodents in the structure and physiologic functions of the brain have resulted in difficulties in reproducing the selective vulnerability of specific neurons or circuits in these mouse models (Chan, 2013; Kishi et al. 2014). In addition, the small-sized brains of rodents are difficult to be analyzed anatomically or functionally in detail by *in vivo* imaging techniques such as MRI or positron emission tomography (PET). These limitations have resulted in the failure to predict the efficacy of clinical trials in human patients from the experimental findings obtained from rodent models of neurodegenerative diseases, lessening the preclinical value of rodent models (Doody et al. 2013, 2014; Salloway et al. 2014). Therefore, the establishment of clinically relevant models of neurodegenerative diseases using nonhuman primates is imperative for accelerating our understanding of pathophysiological mechanisms and the development of clinically applicable therapies (Morton and Howland, 2013; Howland and Munoz-Sanjuan, 2014).

Various nonhuman primate models of human diseases using the chimpanzee, rhesus macaque, cynomolgus macaque, common marmoset, or common squirrel monkey have been reported (Chan, 2013). Among them, the marmoset, a small, nonendangered nonhuman primate, offers many advantages regarding its reproductive features and small body size. Marmosets routinely ovulate multiple oocytes per ovarian cycle, have a short gestation period, and reach sexual maturity at ∼1 year of age (Kishi et al. 2014). Their small body size also enables us to handle them with ease, which translates into lower caging and feeding costs. Furthermore, reproductive technologies for the marmoset, such as embryonic stem cells (Sasaki et al. 2005), transgenic animals with transgene germline transmission (Sasaki et al. 2009), and induced pluripotent stem cells (Tomioka et al. 2010), have been rapidly developed in just the past decade. These advantages of the marmoset compared with other nonhuman primates may provide better outcomes once it has been used in translational research.

In this study, we successfully generated a marmoset model of the polyglutamine (polyQ) diseases, which are a group of inherited neurodegenerative diseases including HD and various types of SCA. The polyQ diseases are caused in common by an expansion mutation of the polymorphic CAG repeat (>35–40 repeats) encoding the glutamine stretch in each disease-causative gene. One of the distinctive features of the polyQ diseases is a tight genotype–phenotype correlation between the number of CAG repeats and the age of disease onset, which makes it a promising avenue to establish a symptomatic transgenic marmoset model by genetic engineering. Considering that various neurodegenerative diseases including AD, PD, and the polyQ diseases share a common pathomechanism of abnormal accumulation of misfolded proteins (Nagai and Popiel, 2008; Costa and Paulson, 2012), our success in the establishment of a symptomatic transgenic marmoset line accompanied by abnormal protein inclusions and neurodegeneration opens new avenues toward elucidating the pathomechanism of and developing therapeutic approaches for various neurodegenerative diseases.

## Materials and Methods

### Animals

All animal experiments were approved by the ethics committee for primate research of the National Center of Neurology and Psychiatry in Japan. All experiments were conducted in accordance with institutional guidelines and the National Research Council’s Guide for the Care and Use of Laboratory Animals.

### Construction of the mutant ataxin 3-120Q vector

**Figure 1.**
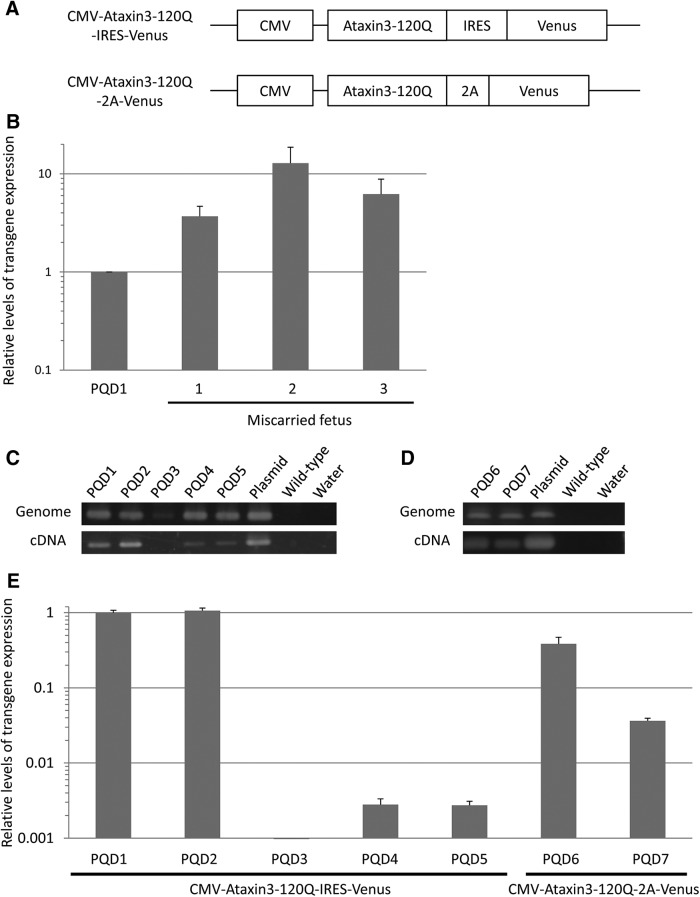
Generation of seven ataxin 3-120Q transgenic marmosets. ***A***, Two types of constructs (CMV-ataxin 3-120Q-IRES-Venus and CMV-ataxin 3-120Q-2A-Venus) were introduced into marmoset embryos. ***B***, Relative levels of transgene expression in PQD1 and three miscarried fetuses are shown. The relative gene expression level in PQD1 was set as 1. ***C***, ***D***, Genomic-PCR (top) and RT-PCR (bottom) analyses of each transgenic marmoset transduced with the CMV-ataxin 3-120Q-IRES-Venus construct (***C***) or the CMV-ataxin 3-120Q-2A-Venus construct (***D***). The results in seven transgenic marmosets (PQD1-7), a positive control (Plasmid), and negative controls (Wild-type and Water) are shown. ***E***, Relative levels of transgene expression in ear fibroblast cells of each transgenic marmoset are shown. The relative gene expression level in PQD1 was set as 1.

The expansion mutation of CAG repeats within the ataxin 3 gene (more than ∼52 repeats; normal 12–41 repeats) is causative for SCA3/Machado-Joseph disease, which is the most common subtype of autosomal dominant SCAs (Costa and Paulson, 2012). The full-length human ataxin 3 gene with 120 CAGs (ataxin 3-120Q) and a marker gene coding Venus protein were linked by an internal ribosomal entry site (IRES) or a self-cleaving 2A peptide (2A) sequence (Fig. 1*A*). The resultant constructs were inserted into a self-inactivating lentiviral vector carrying the CMV promoter (Riken) using Gateway technology (Invitrogen). CAA triplets were inserted every 30 CAG repeats to avoid CAG repeat length mutations.

### Lentiviral vector preparation and transduction

The lentiviral vectors were produced as previously described (Sasaki et al. 2009). Briefly, lentiviruses were generated by cotransfection of lentiviral vectors coding for CMV-ataxin 3-120Q-IRES-Venus or CMV-ataxin 3-120Q-2A-Venus, pCAG-HIVgp (Riken), and pCMV-VSV-G (Riken) into 293FT packaging cells (Invitrogen). The medium containing viral particles was spun at 4°C and 50,000 × *g* for 2 h, and the viral pellet was then resuspended in BlastAssist medium (Origio) in 1/1000 of the volume of the original lentiviral vector supernatant. A high titer (1 × 10^9^ to 1 × 10^11^ transducing units/ml) of lentiviral vectors was used for the subsequent gene transfer. Four-cell to morula-stage embryos were placed in M2 medium (Sigma) supplemented with 0.1 M sucrose, and the viruses were injected into the perivitelline space of embryos using Eppendorf FemtoJet Express (Eppendorf). The embryos were subsequently cultured in BlastAssist medium until embryo transfer.

### Embryo collection and transfer

To determine the ovarian cycle of female marmosets, 100 µl of blood was taken from the femoral vein 11 and 13 days after injection of the prostaglandin F2 alpha analog cloprostenol (Cloprostenol C; Fujita Pharmaceuticals), and ovulation days were determined by measuring plasma progesterone concentrations using an enzyme immunoassay (AIA-360; Tosoh Corp.). The day of ovulation (d 0) was designated as the day before the increase in progesterone concentrations from basal levels to >10 ng/ml. The animals were subjected to embryo collection on d 4–7 and embryo transfer on d 2–5. Embryo collection and transfer were performed by nonsurgical methods as previously described (Ishibashi et al. 2013a, b). Briefly, for embryo collection, a blunt tapered 28-G inner stainless steel cannula and a blunt tapered 19-G outer stainless steel cannula were inserted into the uterus with the aid of ultrasonography. While compressing the oviducts, 4 ml of flushing medium was injected through the inner cannula into the uterus. The perfused medium was collected in a dish as it dripped from the outer cannula. For embryo transfer, one or two embryos were transferred to the uterus of each surrogate mother using the same instruments as for embryo collection. After embryo transfer, the recipients were monitored for pregnancy by ultrasound imaging once a month until delivery.

### Establishment of ear fibroblast cell lines of transgenic marmosets

A small piece of tissue from the ear was collected, minced with scissors, and incubated in PBS containing 1 mg/ml of collagenase (Wako) for 30 min at 37°C. Cells were washed twice by centrifugation and cultured in DMEM containing 10% fetal bovine serum (Gibco).

### DNA/RNA extraction and reverse transcription

DNA/RNA was isolated from ear fibroblast cells or other tissues of the marmosets using the AllPrep DNA/RNA minikit (Qiagen) according to the manufacturer’s instructions. First-strand cDNA was synthesized from RNA using the ReverTra Ace qPCR RT Master Mix with gDNA Remover (Toyobo). As a negative control, RNA was allowed to react with the cDNA synthesis reaction mixture in the absence of reverse transcriptase.

### PCR analysis

To detect the mutant ataxin 3 gene, the CMV forward (5′-GTGGATAGCGGTTTGACTCACG-3′) and ataxin 3 reverse (5′-TGTTGAGCACAAAGTGAGCCTTC-3′) primers were used. To detect the β-actin gene, the β-actin forward 1 (5′-AACTGGGACGACATGGAGAAGATC-3′) and reverse 2 (5′-GTAGCACAGCTTCTCCTTGATGTC-3′) primers were used. Genomic DNA and cDNA were subjected to PCR for 35 cycles of 98°C for 10 s, 64°C for 30 s, and 72°C for 30 s using EmeraldAmp PCR Master Mix (Takara) according to the manufacturer’s instructions. For genomic-PCR of embryos, which were named PQD (for polyQ diseases), each embryo was collected in 10 µl of distilled water and boiled at 98°C for 5 min. Genomic DNA was subjected to PCR for 35 cycles of 98°C for 10 s, 64°C for 10 s, and 68°C for 30 s using KOD-Plus-Neo (Toyobo) supplemented with 40 ng/µl of T4 gene 32 protein (Wako) at a final concentration according to the manufacturer’s instructions.

### Real-time PCR analysis

To quantify the relative expression of transgenes, the Venus forward (5′-TCTTCAAGGACGACGGCAACTAC-3′) and reverse (5′-GTTGTGGCTGTTGTAGTTGTACTCC-3′) and GAPDH forward (5′-TGACAACAGCCTCAAGATCG-3′) and reverse (5′-ACGGTGGTCATGAGTCCTTC-3′) primers were used. cDNA was subjected to real-time PCR for 40 cycles of 95°C for 5 s and 60°C for 30 s using SYBR Premix Ex Taq II (Takara) according to the manufacturer’s instructions. All real-time PCR results were normalized with GAPDH.

### Western blot analysis

Total protein was extracted from ear fibroblast cells or other tissues from the marmosets using DUALXtract (Dualsystems Biotech). The protein extract was boiled with loading dye before loading onto 10% polyacrylamide gels (Bio-Rad). After electrophoresis, proteins were transferred onto a PVDF membrane (Bio-Rad) using Trans-Blot (Bio-Rad) followed by blocking in Tris-buffered saline containing 2.5% bovine serum albumin for 1 h. The membrane was incubated with the mouse monoclonal 1C2 primary antibody (1:3000; Millipore) followed by a peroxidase-conjugated mouse IgG secondary antibody (1:3000; Cell Signaling Technology), and then the proteins were detected with the ECL prime Western Blotting Detection Reagent (GE Healthcare).

### Sequencing of CAG repeats

To detect the presence of CAG repeats within the transgene, the CAG forward 1 (5′-GCTAAGTATGCAAGGTAGTTCCAG-3′) and reverse 6 (5′-GTCTTCTTCACTCATAGCATCACC-3′) primers were used. The cDNA was subjected to PCR for 35 cycles of 98°C for 10 s and 68°C for 30 s using Tks Gflex DNA Polymerase (Takara) according to the manufacturer’s instructions. Then, the PCR products were sequenced using CAG forward 1, CAG forward 2 (5′-GGAAGAGACGAGAAGCCTAC-3′), CAG reverse 5 (5′-TCCCAAGTGCTCCTGAACTG-3′), and CAG reverse 6 primers to determine the number of CAG repeats. The original plasmids were used as positive controls.

### Fluorescent *in situ* hybridization (FISH) analysis

FISH analyses were performed according to the previous report (Pinkel et al. 1986) at the Chromocenter (Tottori, Japan). Briefly, ear fibroblast cells of transgenic marmosets were cultured for 4 h with 0.15 µg/ml of demecolcine (Sigma) and suspended in 0.075 M KCl for 15 min, followed by fixation in methanol/acetic acid (3:1, v/v). The fixed metaphase spreads of the cells were subjected to FISH analysis using biotin-labeled probes for the transgenes, and the bound label was detected using Alexa Fluor 488 streptavidin (Life Technologies). Chromosomal DNA was counterstained with DAPI (Sigma). The images were captured using the Zeiss Axio Imager 2. The FISH signals were analyzed visually by counting ∼20–60 cells per each transgenic marmoset, and chromosomes in which the signal was reproducibly detected in multiple cells were regarded as positive.

### Spontaneous motor activity

The spontaneous motor activities of each marmoset after weaning at 3 months of age were detected and recorded every minute with an infrared sensor (AMN32111, Panasonic). The results were totaled for a day to analyze the amount of spontaneous motor activity and for every hour to analyze diurnal rhythms in the activity.

### Bar grip test

The neuromuscular function of the upper extremities of the marmosets was evaluated using the bar grip test (Smith et al. 1995) after weaning at 3 months of age. The experimenter allowed the marmoset to grasp the bar (bar diameter of 0.7 mm in a 6 × 6-mm grid pattern, MK-380M, Muromachi Kikai) with its forelimbs while suspending the marmoset by its tail, and then pulled the tail with a steadily increasing horizontal force. The test was performed three times on each test day, and the maximal grip strength was recorded.

### Magnetic resonance imaging

MRI images of the brain were obtained from six marmosets; PQD1, 3, 4, and 5 at 13.5 months of age, and PQD6 and 7 at 9.5 months of age. The marmoset’s head was fixed in an MRI-compatible stereotaxic frame and scanned using a four-channel array coil on a 3-T MRI (Siemens Trio). A three-plane localizer image was obtained to ensure correct positioning of the target images [repetition time (TR), 100 ms; echo time (TE), 5 ms; flip angle (FA), 40°; field of view (FOV), 120 mm; slice thickness, 3 mm]. Subsequently, three-dimensional T1-weighted images were taken using a magnetization prepared rapid gradient echo sequence (TR, 2300 ms; TE, 2.8 ms; inversion time, 1000 ms; FA, 12°; FOV, 67 mm; image matrix, 192; in-plane voxel size, ∼0.3 mm).

### Fourth ventricle measurement

T1-weighted MRI images were analyzed using ZioTerm 2009, version 2.0.0.4 (Ziosoft). Using multiplanar reconstruction, tridimensional images were loaded and tilt was corrected manually if necessary to achieve symmetry in the coronal plane. Subsequently, the fourth ventricle was located using the level where the paraflocculus posterior area was larger as the reference point (stereotaxic reference: interaural −2.65 mm; Hardman and Ashwell, 2012). After each measurement, a snapshot was taken and all images were compared with confirm the consistency of the selected plane. To standardize the fourth ventricle measurements in relation to the cerebrum, axial MRI T1-weighted images were used. The University of Texas Health Science Center at San Antonio Image Tool for Windows software version 3.0 was used to measure the cerebral area of sequential images from the top of the head to the area above the eyeballs of each marmoset. The largest area was selected, and data were expressed as the ratio of the area of the fourth ventricle to that of the cerebrum.

### Histochemical analysis of transgenic marmoset tissues

Postmortem brains, spinal cords, peripheral nerves from the upper and lower limbs, and quadriceps femoris muscle of PQD1 and 2 were fixed in 4% paraformaldehyde overnight, embedded in paraffin, and sectioned at a thickness of 5 µm. The sections were stained with hematoxylin and eosin (HE) using standard procedures. Immunohistochemistry was performed with the Ventana XT Discovery (Roche) and the I-View DAB Universal Kit (Roche) according to the manufacturer’s instructions. The following antibodies were used: monoclonal anti-1C2 (1:5000, Merck Millipore), polyclonal anti-ubiquitin (1:1000, Dako), monoclonal anti-GFAP (1:500, Agilent), and polyclonal anti-calbindin (1:16,000, Swant). Nuclei were stained with hematoxylin. For peripheral nerve analysis, samples were postfixed with 1% osmium tetroxide, embedded in epoxy resin, sectioned at 1-µm thickness, and stained with toluidine blue. Stained sections were examined under an Eclipse 90i microscope (Nikon).

### Histochemical analysis of tibialis anterior muscle

Skeletal muscle tissue of PQD1 and 2 was processed for pathologic analysis as reported previously (Malicdan et al. 2009). Briefly, serial 10-µm-thick cryosections were stained with HE and modified Gomori trichrome, for acid phosphatase activity and NADH reductase activity. Stained sections were observed under a BX51 microscope (Olympus), and digitized images were acquired for pathologic analysis using a DP70 digital camera (Olympus).

### Biochemical analysis

Creatine kinase activity in the plasma of PQD2 and four wild-type marmosets was measured using Cicaliquid CK (Kanto Chemical) according to the manufacturer’s protocol. The plasma of PQD1 was not successfully obtained at euthanasia.

### Production of second-generation transgenic marmoset from PQD1 sperm

Second-generation transgenic marmoset embryos were produced as described previously (Tomioka et al. 2012; Takahashi et al. 2014). Briefly, PQD1 was induced to ejaculate using a vibrator, and the sperm were collected in TYH medium (Mitsubishi Kagaku Iatron). Germinal vesicle-stage oocytes of wild-type marmosets were retrieved from follicles (500 µm in diameter) by puncture and cultured for 30 h in POM (Wako) supplemented with 100 mIU/mL of follicle-stimulating hormone. Maturated oocytes were fertilized with PQD1 sperm in M2 medium (Sigma-Aldrich) by intracytoplasmic sperm injection using a micromanipulator (Narishige) with the piezo drive system (Primetech). The embryos were subsequently cultured in TYH medium until embryo transfer.

## Results

### Production of transgenic marmosets expressing expanded CAG repeats

Full-length human ataxin 3 cDNA with 120 CAGs (ataxin 3-120Q) was inserted into a self-inactivating lentiviral vector carrying the CMV promoter. We used the lentiviral vector and the CMV promoter to induce efficient integration and high expression of the transgene (Sasaki et al. 2009). Highly efficient transgene integration enables successful transgenesis with a smaller number of embryos, and lentiviral transfection is less invasive than pronucleus injection for DNA delivery to the fertilized eggs or embryos (Chan, 2013). A high titer of lentiviral vector carrying CMV-ataxin 3-120Q-IRES-Venus or CMV-ataxin 3-120Q-2A-Venus (Fig. 1*A*) was injected into 29 and 37 embryos, followed by embryo transfer to 17 and 23 surrogate mothers, respectively (Table 1). A total of 14 surrogates became pregnant, and five surrogates delivered a total of seven offspring. These offspring showed no neurologic symptoms at birth. The remaining nine surrogates miscarried midpregnancy after observation of fetal cardiac arrest or heartbeat weakness by ultrasound imaging. This high abortion rate indicates that the expression of ataxin 3-120Q exerts strong cytotoxicity during fetal development, supported by a 3.7- to 12.8-fold higher level of the transgene expression in the miscarried fetuses than that in PQD1 (Fig. 1*B*). Considering that ear fibroblast cells from all of the surviving offspring showed no visible Venus fluorescence under UV light, only those offspring with lower transgene expression levels appeared to have survived until birth.

**Table 1.** Production rates of transgenic marmosets

	CMV-Ataxin3-120Q-IRES-Venus	CMV-Ataxin3-120Q-2A-Venus	Total
Number of embryos transferred to surrogates	29	37	66
Number of surrogates	17	23	40
Number of pregnancies	7	7	14
Number of deliveries	3	2	5
Number of births (percentage of births per embryos transferred)	5 (17.2)	2 (5.4)	7 (10.6)
Number of transgenic animals (percentage of transgenic animals per birth)	5 (100)	2 (100)	7 (100)

A high titer of lentiviral vector was injected into a total of 66 embryos, followed by embryo transfer to 40 surrogate mothers. A total of 14 surrogates became pregnant, and five surrogates delivered seven offspring.

### Molecular biological analyses of transgenic marmosets

Transgene integration and expression were examined in ear fibroblast cells from each offspring. All seven offspring carried the ataxin 3-120Q transgene, and six of seven offspring (except PQD3) expressed the transgene (Fig. 1*C*, *D*). In particular, three transgenic marmosets, PQD1, 2, and 6, expressed higher levels of the transgene than the other transgenic marmosets, as revealed by quantitative real-time PCR analyses (Fig. 1*E*). Although all marmosets except PQD3 carried the ataxin 3-120Q transgene, transgenes with other CAG repeat lengths such as 80 and 43 CAG repeats were also found to be integrated in PQD5 and 7, respectively (Table 2). FISH analyses using ear fibroblast cells from PQD1, 2, and 6 showed that each marmoset had several integration sites and cellular mosaicism; positive FISH signals from the transgenes were reproducibly detected in chromosomes 1, 4, and 17 in PQD1; chromosomes 1, 17, and 22 in PQD2; and chromosomes 7 and 22 in PQD6, with patterns that sometimes varied among different cells (Table 2).

**Table 2.** Summary of the characteristics of the seven transgenic marmosets

ID	Sex	CAG repeats	Chromosomes with transgene integration	Disease onset	Disease progression
PQD1	Male	120	1, 4, 17	4 months	Moderate
PQD2	Female	120	1, 17, 22	3 months	Severe
PQD3	Female	ND			
PQD4	Female	120			
PQD5	Female	120, 80			
PQD6	Female	120	7, 22	4 months	Mild
PQD7	Male	120, 43			

ND, not determined.

Although all marmosets except PQD3 carried the ataxin 3-120Q transgene, transgenes with other CAG repeat lengths were also found to be expressed in PQD5 and 7, respectively. PQD1, 2, and 6 had several integration sites and cellular mosaicism and developed neurologic symptoms of varying degrees at 3–4 months after birth.

### Behavioral analyses of transgenic marmosets

**Figure 2.**
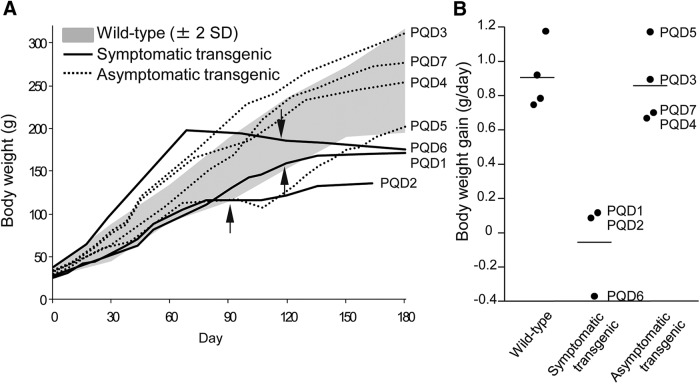
Age-dependent changes in body weight and body weight gain. ***A***, Age-dependent changes in the body weight of wild-type marmosets, symptomatic transgenic marmosets (PQD1, 2, and 6), and asymptomatic transgenic marmosets (PQD3, 4, 5, and 7). Arrows indicate the onset of neurologic symptoms of the three symptomatic transgenic marmosets. Averages ± 2 SD of body weight in wild-type marmosets (*n* = 4) are indicated as gray areas. ***B***, Body weight gain of wild-type marmosets, symptomatic transgenic marmosets, and asymptomatic transgenic marmosets at 5–6 months of age. Each symbol represents an individual marmoset. Horizontal bars indicate the group average.

**Figure 3.**
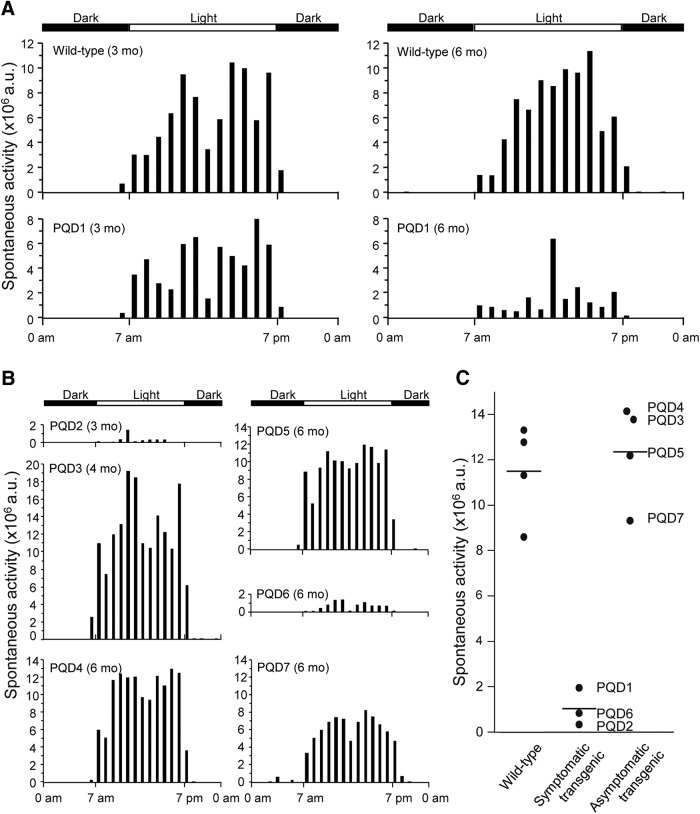
Transgenic marmosets show progressive neurologic symptoms. ***A***, Age-dependent changes in 24-h spontaneous activity of a wild-type marmoset and PQD1 transgenic marmoset at 3 and 6 months. ***B***, 24-h spontaneous activity of PQD2 at 3 months, PQD3 at 4 months, and PQD4–7 at 6 months of age. ***C***, 24-h spontaneous activity levels of PQD1 at 5–6 months of age, PQD2 at 3 months, and PQD6 at 5–6 months of age, compared with those of wild-type marmosets and asymptomatic transgenic marmosets at 5–6 months of age. Each symbol represents an individual marmoset. Horizontal bars indicate the group average.

**Figure 4.**
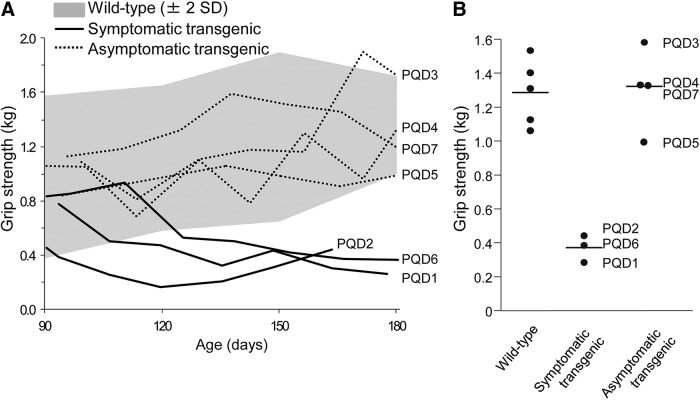
Age-dependent changes in grip strength. ***A***, Age-dependent changes in grip strength of wild-type marmosets, symptomatic transgenic marmosets (PQD1, 2, and 6), and asymptomatic transgenic marmosets (PQD3, 4, 5, and 7). Averages ± 2 SD of grip strength in wild-type marmosets (*n* = 4) are indicated as gray areas. ***B***, Grip strength of wild-type marmosets, symptomatic transgenic marmosets, and asymptomatic transgenic marmosets at 5–6 months of age. Each symbol represents an individual marmoset. Horizontal bars indicate the group average.

Although all animals showed no symptoms at birth and grew normally, PQD1, 2, and 6, which expressed ataxin 3-120Q at higher levels, developed neurologic symptoms of varying degrees at 3–4 months after birth (Table 2). In the early stages of the disease, these three transgenic marmosets often slipped down from the grids of the cage, and their jumping ability was slightly but noticeably decreased, and the symptoms progressed to a severe movement disability accompanied by contractures of the legs and arms in the later stages. These symptomatic transgenic marmosets showed gradual decreases in body weight gain and almost no further weight gain beyond 3–4 months of age (Fig. 2*A*, *B*). The levels of body weight gain were significantly lower than those of wild-type marmosets and asymptomatic transgenic marmosets at 5–6 months of age (Fig. 2*B*; *p* < 0.01). Spontaneous activity monitoring revealed a progressive decline in activity in PQD1 compared with a wild-type marmoset, without apparent diurnal rhythm alterations (Fig. 3*A*). Spontaneous activity levels of PQD2 and 6 after onset of symptoms were also significantly lower than those of wild-type marmosets and asymptomatic transgenic marmosets (Fig. 3*B*, *C*; *p* < 0.01). Grip strengths of PQD1, 2, and 6 gradually decreased (Fig. 4*A*) and were significantly lower than those of wild-type marmosets at 5–6 months of age (Fig. 4*B*; *p* < 0.01). As shown in these figures, all behavioral measurements as well as the body weight of asymptomatic transgenic marmosets were comparable with that of wild-type marmosets. For example, the body weight of asymptomatic transgenic marmosets showed a progressive increase throughout the whole period of measurement (Fig. 2*A*), and the level of body weight gain was not different from that of the wild-type (Fig. 2*B*). Similarly, no significant difference was found in the spontaneous activity (Fig. 3*C*) or grip strength (Fig. 4*B*).

### Molecular biological analyses of symptomatic transgenic marmosets

**Figure 5.**
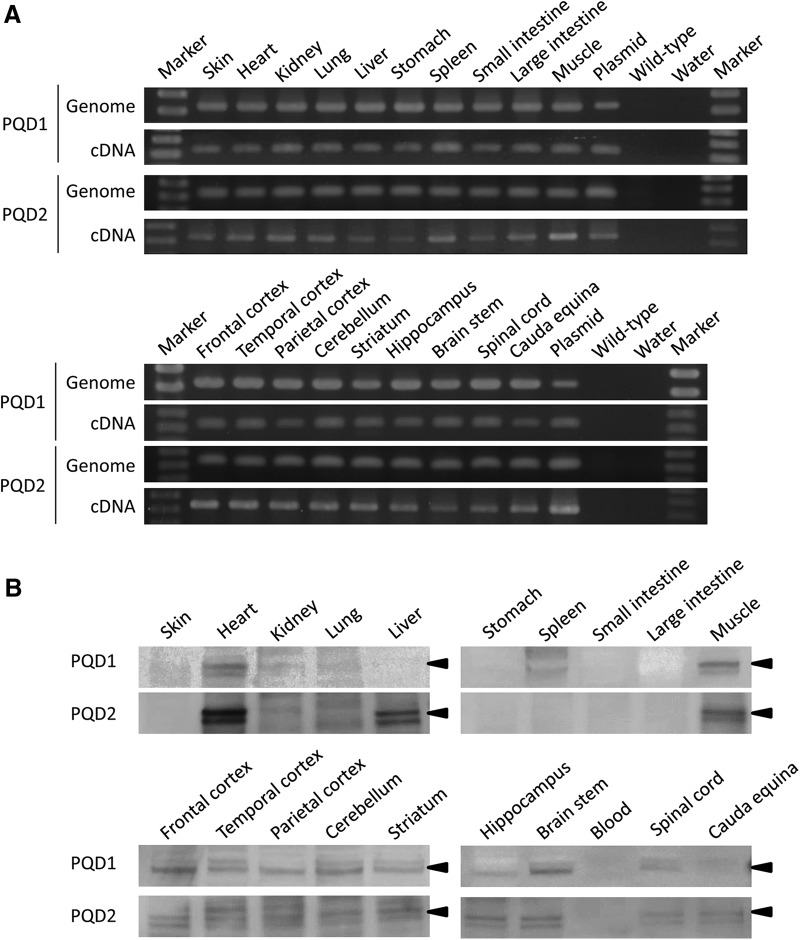
Expression of ataxin 3-120Q in transgenic marmosets PQD1 and 2. ***A***, ***B***, Genomic-PCR (top) and RT-PCR (bottom) analyses (***A***) and Western blot analysis (***B***) of the major organs and various brain regions of PQD1 and 2. A positive control (Plasmid) and negative controls (Wild-type and Water) were also included. Arrowheads indicate the position of the ataxin 3-120Q band.

Because of their severe difficulty in eating by themselves and their progressive body weight loss, PQD1 and PQD2 were euthanized at 13.5 and 5.5 months of age, respectively, and were subjected to biochemical and pathologic analyses. Integration and expression of the mutant ataxin 3 transgene in the major organs of PQD1 and 2 were examined. Genomic and RT-PCR analyses confirmed integration and expression of the transgene in all analyzed organs of both PQD1 and 2 (Fig. 5*A*). Western blot analyses revealed expression of the expanded polyQ protein in the heart, kidney, lung, spleen, skeletal muscle, and various brain regions in PQD1 and the heart, liver, skeletal muscle, and various brain regions in PQD2 (Fig. 5*B*).

### Pathologic analyses of symptomatic transgenic marmosets

**Figure 6.**
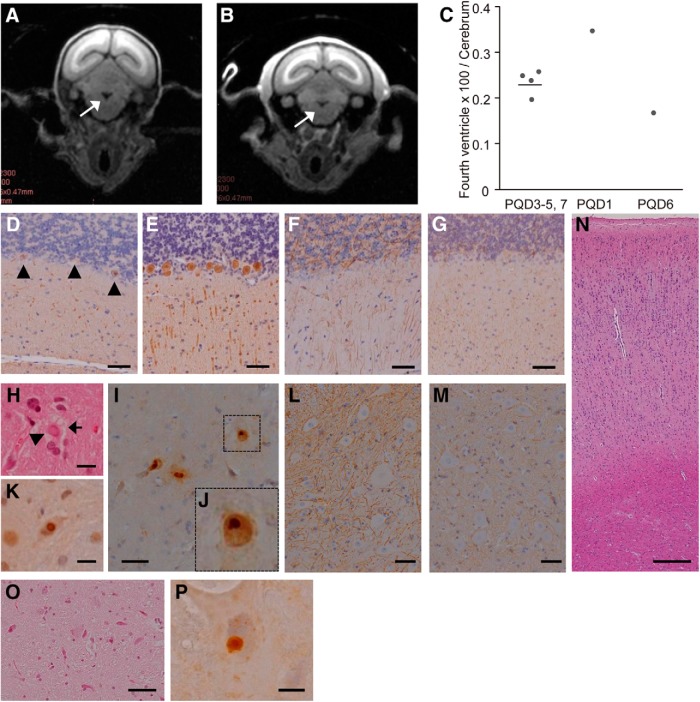
Cerebellar atrophy and neurodegeneration accompanied by polyQ protein inclusions in the brains of symptomatic transgenic marmoset PQD1. ***A***, ***B***, Coronal MRI sections of the brain of PQD1 (***A***) and asymptomatic marmoset PQD5 (***B***). The arrow indicates the fourth ventricle. ***C***, Quantitative analyses of brain MRIs of transgenic marmosets. The bar indicates the mean of PQD3–5 and 7. ***D***, ***E***, Anti-calbindin staining of the cerebellum of PQD1 (***D***) or a control wild-type marmoset (***E***). Arrowheads indicate degenerated Purkinje cells. ***F***, ***G***, Anti-GFAP staining of the cerebellum of PQD1 (***F***) or a control wild-type marmoset (***G***). ***H–K***, Hematoxylin and eosin (HE; ***H***), anti-1C2 (***I*** and ***J***), and anti-ubiquitin (***K***) staining of the spinal cord of PQD1. In ***H***, the arrow indicates a degenerated anterior horn cell, and the arrowhead indicates an inclusion body. A magnified image of a 1C2-positive inclusion (***I***) is shown in ***J***. ***L***, ***M***, Anti-GFAP staining of the spinal cord of PQD1 (***L***) and a control wild-type marmoset (***M***). ***N***, HE staining of the cerebrum of PQD1. ***O***, ***P***, HE (***O***) and anti-1C2 (***P***) staining of the brainstem of PQD1. Scale bars: 100 µm in ***D–H*** and ***K***; 200 µm in ***G***; 50 µm in ***I***, ***L***, ***M***, and ***O***; 250 µm in N; and 10 µm in ***P***.

Noninvasive MRI analyses were performed on two of the three symptomatic marmosets, PQD1 at 13.5 months of age and PQD6 at 9.5 months of age, and four asymptomatic transgenic marmosets at the ages corresponding to either PQD1 or 6. MRI scanning revealed significant enlargement of the fourth ventricle in one of the symptomatic marmosets, PQD1, compared with asymptomatic transgenic marmosets, suggesting cerebellar atrophy (Fig. 6*A–C*; relative fourth ventricle area: PQD1, 0.348 arbitrary units (au); PQD6, 0.169 au; average of four asymptomatic transgenic marmosets, 0.235 ± 0.049 au). Consistent with the MRI finding, immunohistochemical analysis of PQD1, which was euthanized immediately after the MRI scanning, revealed a marked loss of Purkinje cells (Fig. 6*D*, *E*) accompanied by gliosis (Fig. 6*F*, *G*) in the cerebellum. In addition, degeneration of lower motor neurons and eosinophilic intranuclear inclusions were evident in the anterior horn of the cervical and lumbar spinal cord of PQD1 (Fig. 6*H*). Importantly, these inclusions were immunoreactive for the 1C2 antibody (Fig. 6*I*, *J*), which specifically recognizes the expanded polyQ stretch, and for the ubiquitin antibody (Fig. 6*K*). Mild gliosis was also detected in the spinal cord of PQD1 (Fig. 6*L*, *M*). The cerebrum and brainstem did not show significant pathology (Fig. 6*N*, *O*) except for scarce 1C2-positive inclusions observed in the brainstem (Fig. 6*P*).

**Figure 7.**
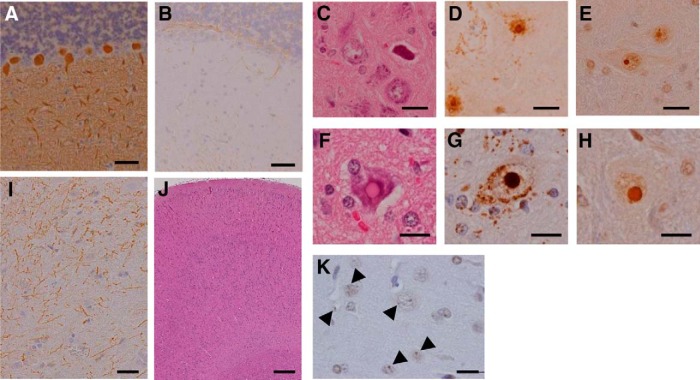
Neurodegeneration and polyQ protein inclusions in the brain and spinal cord of symptomatic transgenic marmoset PQD2. ***A***, ***B***, Anti-calbindin (***A***) and GFAP (***B***) staining of the cerebellum of PQD2. ***C–H***, HE (***C*** and ***F***), anti-1C2 (***D*** and ***G***), and anti-ubiquitin (***E*** and ***H***) staining of the brainstem (***C–E***) and spinal cord (***F–H***) of PQD2. ***I***, Anti-GFAP staining of the spinal cord of PQD2. ***J***, ***K***, HE (***J***) and anti-ubiquitin (***K***) staining of the cerebrum of PQD2. These inclusions (arrowheads in ***K***) were not immunoreactive for the 1C2 antibody. Scale bars: 100 µm in ***A***, ***B***, and ***F–H***; 5 µm in ***C–E***; 50 µm in ***I***; 250 µm in ***J***; and 10 µm in ***K***.

On the contrary, the immunohistochemical analysis of PQD2, on which MRI analysis was not performed, did not show significant cerebellar pathology (Fig. 7*A*, *B*). However, intranuclear eosinophilic and 1C2-positive inclusions similar to those observed in PQD1 were frequently observed in the brainstem (Fig. 7*C*, *D*) and spinal cord (Fig. 7*F*, *G*) of PQD2. These inclusions were also positive for the ubiquitin antibody (Fig. 7*E*, *H*). The spinal cord also showed neurodegeneration (Fig. 7*F*) accompanied by mild gliosis (Fig. 7*I*). Although the cerebrum of PQD2 did not show significant pathology in HE staining (Fig. 7*J*), ubiquitin-positive but 1C2-negative inclusions were observed (Fig.7*K*).

These results suggest that the symptomatic transgenic marmosets PQD1 and 2 successfully recapitulated the common pathologic characteristics of the polyQ disease patients, namely, neurodegeneration and the formation of intranuclear inclusions composed of abnormal protein with polyQ expansion (Table 3).

**Table 3.** Summary of the pathologic findings of PQD1 and 2

	PQD1		PQD2	
	13.5 months		5.5 months	
	Degeneration	Inclusion	Degeneration	Inclusion
Cerebrum	−	−	−	+
Cerebellum	++	−	−	−
Brain stem	−	±	−	+
Spinal cord	++	++	++	++
Peripheral nerves	++	N/E	++	N/E
Skeletal muscles	+++	++	+++	++

Two marmosets among the three symptomatic transgenic marmosets were subjected to immunohistochemical analysis. Note that cerebellar degeneration was observed in PQD1. N/E: not examined.

**Figure 8.**
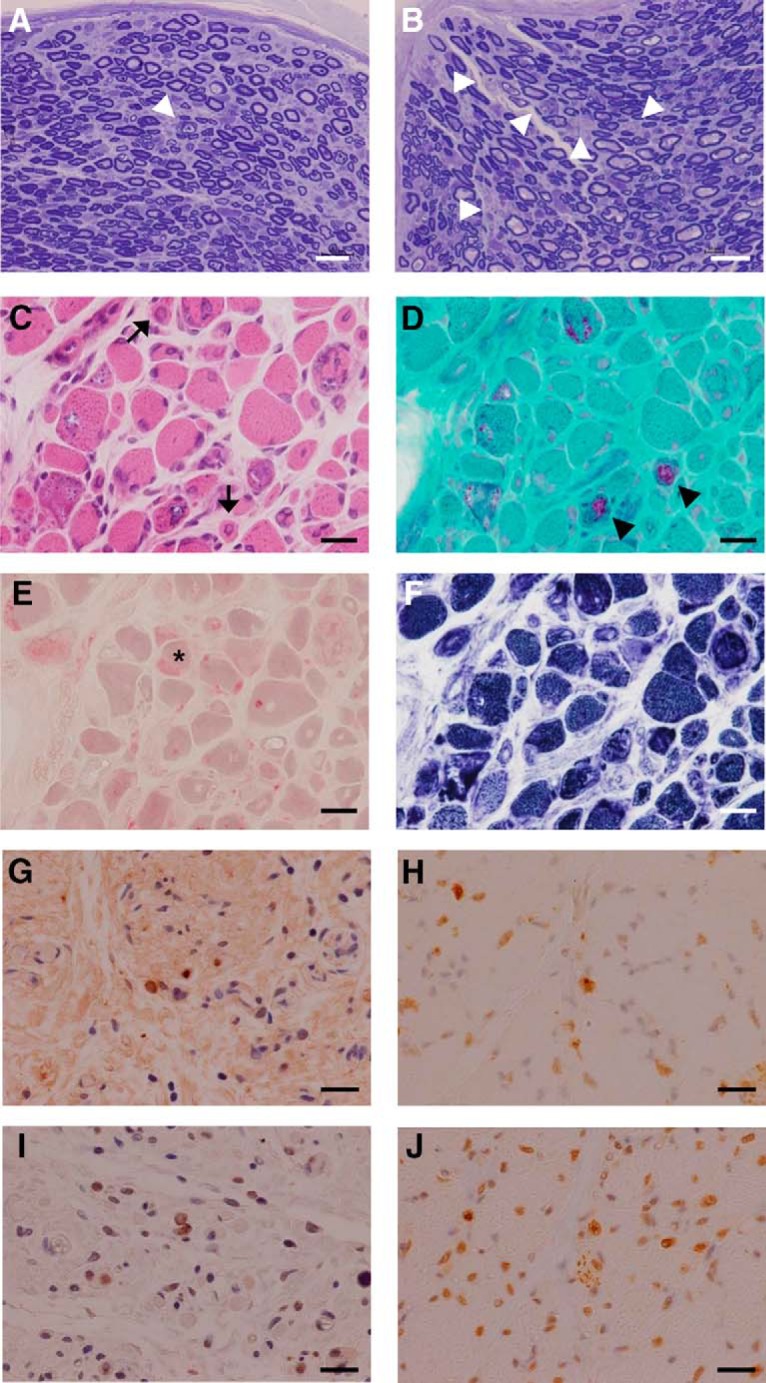
Pathology of the peripheral nerves and muscles of PQD1 and 2. ***A***, ***B***, Toluidine blue staining of the peripheral nerves of the upper limbs of PQD1 (***A***) and PQD2 (***B***). Arrowheads indicate axonal degeneration. ***C–F***, HE (***C***), modified Gomori trichrome (***D***), acid phosphatase activity (***E***), and NADH reductase activity (***F***) staining of the tibialis anterior muscle of PQD2. Marked fiber size variation and endomysial fibrosis were observed. Fibers with centrally placed large nuclei and intranuclear inclusions (arrows in ***C***) or rimmed vacuoles (arrowheads in ***D***) and acid phosphatase–positive fibers (asterisk in ***E***) were also observed. The intermyofibrillar network was disorganized in many fibers (***F***). Dystrophic changes, group atrophy, and fiber-type grouping were not observed. ***G–J***, Anti-1C2 (***G*** and ***I***) and anti-ubiquitin (***H*** and ***J***) staining of the quadriceps femoris muscle of PQD1 (***G*** and ***H***) and PQD2 (***I*** and ***J***). Scale bars: 100 μm in ***A*** and ***B***; 20 μm in ***C–J***.

Our transgenic marmosets also exhibited several additional pathologies in the peripheral nerves and muscles, probably because of widespread expression of the expanded polyQ protein under the CMV promoter. Acute axonal degeneration was observed in the peripheral nerves of the upper and lower limbs of PQD1 and 2 (Fig. 8*A*, *B*). PQD1 and 2 also exhibited muscular pathology consistent with chronic myopathic changes caused by the ataxin 3 protein with expanded polyQ stretch (Fig. 8*C–F* and Table 3). Degenerating myofibers with eosinophilic intranuclear inclusions were observed in PQD2 (Fig. 8*C*). The muscles of PQD1 exhibited severe degeneration accompanied by marked fat deposition, and thus it was difficult to detect these changes, including eosinophilic inclusions using cryosections of the tibialis anterior muscle. Immunohistochemical analysis of the PFA-fixed sections with the 1C2 antibody, however, demonstrated abundant accumulation of the inclusions with expanded polyQ protein in both PQD1 and 2 in the quadriceps femoris muscles (Fig. 8*G*, *H*). These inclusions were also positive for the ubiquitin antibody (Fig. 8*I*, *J*). The creatine kinase level of PQD2 was mildly elevated (226 IU/l) compared with those of wild-type marmosets (88.2 ± 83.6 IU/l, *n* = 4).

### Germline transmission of the transgene

**Figure 9.**
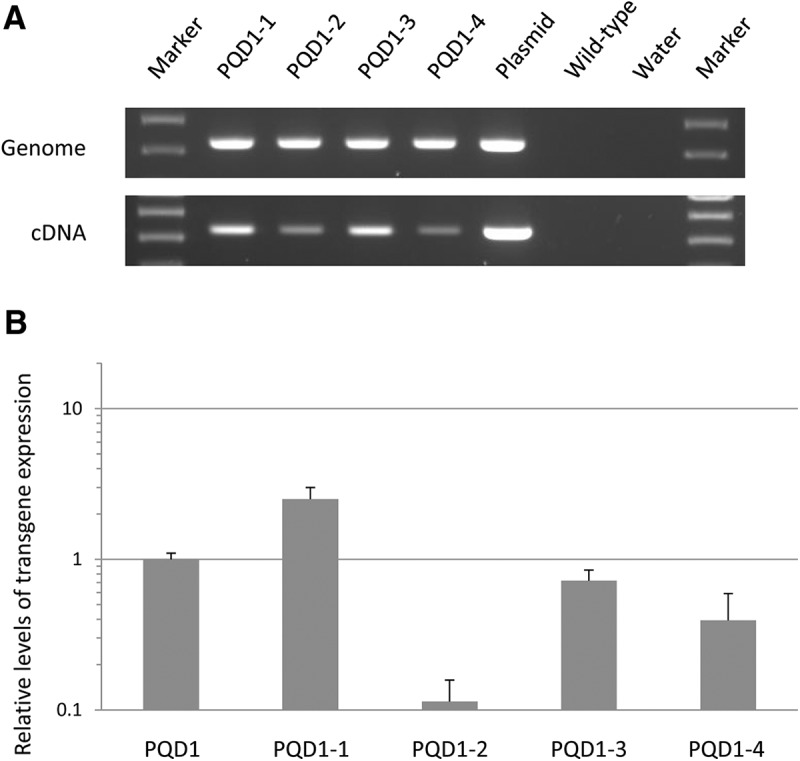
Germline transmission of the ataxin 3-120Q transgenes. ***A***, Genomic-PCR (top) and RT-PCR (bottom) analyses in second-generation transgenic offspring. The results in PQD1-1, 1-2, 1-3, and 1-4, a positive control (Plasmid), and negative controls (Wild-type and Water) are shown. ***B***, Relative levels of transgene expression in ear fibroblast cells of each second-generation transgenic offspring are shown. The relative gene expression level in PQD1 was set as 1.

To obtain second-generation offspring of the transgenic marmosets, the ejaculated sperm of PQD1 was collected after sexual maturity at the age of 11 months and injected into the oocytes of a wild-type marmoset by intracytoplasmic sperm injection. A total of 40 embryos were transferred to 17 surrogate mothers. As a result, four offspring (named PQD1-1, 1-2, 1-3, and 1-4) were obtained from two surrogate mothers. Transgene integration and expression were examined in ear fibroblast cells from each offspring. All offspring carried and expressed the ataxin 3-120Q transgene with 120 CAG repeats (Fig. 8). Moreover, higher or similar levels of transgene expression in PQD1 were confirmed in PQD1-1 and 1-3, as revealed by quantitative real-time PCR analyses (Fig. 9*B*). FISH analyses using ear fibroblast cells showed that transgenes were integrated into chromosomes 1, 3, 10, and 16 in PQD1-1; chromosomes 4 and 16 in PQD1-2; and chromosomes 3 and 22 in PQD1-3 and 1-4. These results suggested that the transgene inserted chromosomes were different between PQD1 and each second-generation offspring, which is considered to be caused by mosaicism in PQD1.

## Discussion

A significant milestone in modeling human disease in nonhuman primates using the transgenic technique is the earlier report of transgenic HD rhesus monkey models that express the polyQ-expanded huntingtin gene (Yang et al. 2008). Although these transgenic monkeys showed some polyQ-disease–like features, including intranuclear inclusions in the brain, their symptoms appeared immediately after birth, and unfortunately, the symptomatic animals died without germline transmission of the transgenes. In a more recent study, the same group has succeeded in producing transgenic HD rhesus monkey models exhibiting slower onset and progression with the onset of behavioral phenotypes at 8 or 16 months of age (Chan et al. 2014, 2015). Furthermore, they have confirmed germline transmission of the transgene in the second-generation offspring generated from one of the founder monkeys. Unfortunately, however, the number of CAG repeats in the transgene of one of the two offspring obtained in the study was shortened to 53–61 repeats compared with 72–74 repeats in the founder monkey and the other offspring, likely caused by the instability of the triplet repeat sequence (Moran et al. 2015). Although the transgene was expressed under the control of the human *HTT* promoter in this founder monkey and thus is expected to recapitulate the late onset and gradual progression of adult HD, establishing symptomatic HD monkey model colonies in a time-efficient manner for future studies from this founder monkey could be challenging.

In the present study, we successfully generated a transgenic marmoset model of the polyQ diseases with no symptoms at birth, which exhibited progressive motor impairment within several months after the disease onset at 3–4 months of age. To produce transgenic marmoset models that are expected to develop neurologic symptoms at an early age, we decided to use ataxin 3 with a hyperexpanded 120 CAG repeat, as well as the lentiviral vector carrying CMV promoter, which is the only method established so far and currently accepted to generate transgenic marmosets (Sasaki et al. 2009). Hyperexpansion of the CAG repeat above the human disease range (52–86 repeats) has been widely applied to reproduce neurodegenerative diseases in short-lived rodent models, because the number of CAG repeats is inversely correlated with disease onset in the polyQ diseases (Davies et al. 1997; Watase et al. 2002; Boy et al. 2010). In addition, because triplet repeat sequences are known to show instability during DNA replication (Lenzmeier and Freudenreich, 2003), CAA triplets that also encode glutamine were introduced every 30 CAG repeats to avoid the mutation. As a result, all the transgenic marmosets analyzed in our study were confirmed to carry a transgene with the original length of 120 CAG repeats, although some variations in the CAG repeat length were found in PQD5 and 7 (Fig. 1*D*). Through these technical approaches, we successfully generated transgenic marmosets that developed progressive neurologic symptoms within a practical time window as experimental animal models. In addition, it is notable that all second-generation transgenic offspring also carried the transgene with 120 CAG repeats, showing that the transgene was steadily transmitted to offspring.

In this study, two of three symptomatic transgenic marmosets underwent pathologic analyses and were found to have developed polyQ protein inclusions or degeneration in various regions, including brains, spinal cords, peripheral nerves, and muscles. This result is in line with previous studies in transgenic HD rhesus monkey models (Yang et al. 2008; Chan et al. 2015) that exhibited polyQ protein inclusions or degeneration in the cortex and striatum and suggests that the expression of mutant protein with expanded polyQ stretch resulted in these pathologic changes leading to abnormal motor phenotypes in both marmosets and rhesus monkeys. It is of note that the formation of inclusions composed of misfolded proteins and degeneration in the nervous system, which are key features shared among various neurodegenerative diseases such as AD, PD, and the polyQ diseases (Jucker and Walker, 2013; Nagai and Minakawa, 2015), were successfully reproducible in two different primate species.

Intriguingly, our marmoset model seems to have exhibited, at least in part, selectivity in the affected tissues similar to those in SCA3 patients, despite the widespread expression of ataxin 3-120Q protein under the CMV promoter. Indeed, the involvement of the cerebellum, the anterior horn of the spinal cord, and the peripheral nerves are often observed in SCA3 patients (Paulson, 2012). It is also known that the age of symptom onset in the juvenile form of SCA3 patients with a long 86 CAG repeat can be as early as 5 years (Zhou et al. 1997; Paulson, 2012), and that in SCA3, patients with homozygous expansions can be as young as 4 years old (Carvalho et al. 2008), resembling the relatively young onset of our transgenic marmoset line expressing hyperexpanded CAG repeats.

However, the strong ubiquitous expression of the mutant ataxin 3 protein via the CMV promoter could have resulted in the subacute disease progression of the motor phenotype and strong myopathic change in our marmosets, both of which are unusual in SCA3 patients. Furthermore, random integration effects of the lentiviral vector, including copy number variation, might have resulted in the variability of the behavioral and pathologic phenotype among our transgenic marmosets. Indeed, the fact that PQD2, which showed the earliest onset and rapid symptom progression, had a 2.8-fold higher transgene expression level compared with PQD6, which showed relatively late onset and slow symptom progression, suggests that the difference in the transgene expression level affected the severity of motor phenotypes in our marmosets, as was previously described in mouse models of SCA3 (Boy et al. 2009). Nevertheless, we used lentivirus-mediated gene transfer, because this has hitherto been the most successful method of producing transgenic nonhuman primates (Chan, 2013), although the establishment of more precise gene editing techniques that enables the generation of better NHP models such as gene knockouts/knockins is awaited for future studies.

Our transgenic marmoset model showing postgrowth disease onset and progressive motor impairment will be useful for various studies on neurodegenerative diseases, including the identification of disease biomarkers as well as the evaluation of the efficacy and metabolic profiles of therapeutic candidates (Morton and Howland, 2013; Howland and Munoz-Sanjuan, 2014). In addition, our MRI analyses suggested cerebellar atrophy in one of the disease-progressed living marmosets, illustrating a potential advantage of the use of nonhuman primate models with a larger brain size than rodents, which allows for more detailed *in vivo* anatomic evaluation (Hikishima et al. 2013). This advantage, when confirmed in the second-generation offspring obtained from our study, will allow us to monitor subtle temporal changes in the brain during disease onset and progression, or even before onset, by high-resolution functional brain imaging analyses such as MRI and PET. Although the symptomatic transgenic marmosets obtained in the first generation were limited in number and showed phenotypic variation, we successfully achieved transgenic offspring from the symptomatic transgenic marmoset. Despite the variation in the integration site in these offspring, possibly because of mosaicism in the founder marmoset, symptom onset in these offspring is anticipated considering the transgene expression comparable to that of the symptomatic founder marmosets in at least two of the four offspring, and the correlation of the transgene expression and symptom onset in the founder generation. We expect that the cohort of these symptomatic marmosets, once established from these offspring, can be used as a colony source and will contribute to future translational research in a timely manner, aid the development of clinically relevant biomarkers, and be used to evaluate the safety and efficacy of therapeutic candidates of polyQ diseases. Our success in modeling the polyQ diseases in common marmosets should accelerate research not only on neurodegenerative diseases, but also on various other human diseases (Chan, 2013; Kishi et al. 2014).

## Synthesis

Reviewing Editor: Leonidas Stefanis, Biomedical Research Foundation of the Academy of Athens Decisions are customarily a result of the Reviewing Editor and the peer reviewers coming together and discussing their recommendations until a consensus is reached. When revisions are invited, a fact-based synthesis statement explaining their decision and outlining what is needed to prepare a revision will be listed below. The following reviewer(s) agreed to reveal their identity: Deniz Kirik, Erwan Bezard.

Both reviewers feel that the study is interesting and has unique attributes, however the presentation of the results is fragmentary and incomplete. A more comprehensive assessment of the behavioral, imaging and neuropathological data is needed on a case by case basis. Further quantification is needed. There has to be a more objective presentation of the advantages of marmosets as experimental species, given the apparent low yield of usable transgenic offspring in the present study. Statements about the correlation with Human disease need to be toned down, in view of obvious discrepancies (for example, marked muscle involvement). Statements about teh significance of the findings need to be toned down as well, given the lack of statistics and quantitative data in many cases. The whole manuscript needs to be presented in a more unbiased fashion, taking into account caveats and limitations. Wherever limited data are available, this should be clearly acknowledged and statements toned down.

In addition:

1. The presentation of the transgene expression data is quite confusing. Data where all animals, including those without phenotype and those with premature termination, need to be presented together, to get a sense of the relative expression. More data are needed about the prematurely terminated embryos, for example are there any morphological, neuropathological data to buttress the idea of excesive transgene expression?

2. More details are needed about the two different constructs utilized (Ataxin3(120Q)-IRES-Venus and Ataxin3(120Q)-2A-Venus), and the resultant transgene expression.

3. More Information is needed for the second generation offspring (integration sites, mosaicism, Number of repeats), and a discussion about the possibility that these animals could be a source of a colony.

4. It would be important to have the data from the other 4 cases available to the readers, even if they have not shown any pathology, apparently due to much lower expression in them.

5. An important part of the justification in using marmosets as the host species is (according to the authors) the significantly better reproductive capacity of these animals. Yet, they state that from 35 implantations they made in trying the show germline transmission they were able to get only 2 off-springs. Is this going to be seen as a high reproductive capacity in the field by experts?

6. Fig 1E could be expanded into a new Table, with as many fields as possible, as this will be vital Information to the reader.

7. As it is not easy to generate these animals, it would be important to know if the already generated lines can be made accessible to the research community at large. The authors should comment on how they can foresee this process established and what needs to be done to reach there.

8. The movie presented does not add any useful Information.
